# Micronutrient Gaps in Three Commercial Weight-Loss Diet Plans

**DOI:** 10.3390/nu10010108

**Published:** 2018-01-20

**Authors:** Matthew G. Engel, Hua J. Kern, J. Thomas Brenna, Susan H. Mitmesser

**Affiliations:** 1 Division of Nutritional Sciences, Cornell University, Ithaca, NY 14853, USA; mge26@cornell.edu (M.G.E.); tbrenna@utexas.edu (J.T.B.); 2 Nature’s Bounty Co., Ronkonkoma, NY 11779, USA; susanmitmesser@nbty.com; 3 Dell Pediatric Research Institute, Dell Medical School, University of Texas at Austin TX, Austin, TX 78723, USA

**Keywords:** weight-loss diets, nutrient density, micronutrient gaps

## Abstract

Weight-loss diets restrict intakes of energy and macronutrients but overlook micronutrient profiles. Commercial diet plans may provide insufficient micronutrients. We analyzed nutrient profiles of three plans and compared their micronutrient sufficiency to Dietary Reference Intakes (DRIs) for male U.S. adults. Hypocaloric vegan (Eat to Live-Vegan, Aggressive Weight Loss; ETL-VAWL), high-animal-protein low-carbohydrate (Fast Metabolism Diet; FMD) and weight maintenance (Eat, Drink and Be Healthy; EDH) diets were evaluated. Seven single-day menus were sampled per diet (*n* = 21 menus, 7 menus/diet) and analyzed for 20 micronutrients with the online nutrient tracker CRON-O-Meter. Without adjustment for energy intake, the ETL-VAWL diet failed to provide 90% of recommended amounts for B_12_, B_3_, D, E, calcium, selenium and zinc. The FMD diet was low (<90% DRI) in B_1_, D, E, calcium, magnesium and potassium. The EDH diet met >90% DRIs for all but vitamin D, calcium and potassium. Several micronutrients remained inadequate after adjustment to 2000 kcal/day: vitamin B_12_ in ETL-VAWL, calcium in FMD and EDH and vitamin D in all diets. Consistent with previous work, micronutrient deficits are prevalent in weight-loss diet plans. Special attention to micronutrient rich foods is required to reduce risk of micronutrient deficiency in design of commercial diets.

## 1. Introduction

About 39% of adults in the world are overweight and 13% are obese [1]. The prevalence of obesity among U.S. adults is as high as 36.5%; that is, more than one in three American adults are obese [2]. Overweight and obesity are associated with increased risk of morbidities including cardiovascular disease, hypertension and type 2 diabetes [3,4] and are ranked as the second leading cause of preventable death in the United States [5]. Weight loss, even at a moderate level of 5–10%, has been reported to reduce risk factors for obesity-associated chronic diseases among overweight and obese individuals [6,7].

In 2014, Americans were estimated to spend US$2.5 billion on weight-loss services [8]. The U.S. weight loss market is projected to grow to US$ 66 billion in 2017 [9]. The etiology of obesity is multifactorial, including both over-consumption of calories and insufficient intakes of micronutrients [10]. Most weight-loss diets restrict calories and macronutrients such as carbohydrates and fats but overlook micronutrient content [11]. A few studies analyzed micronutrient composition of weight-loss diets including Dr. Atkins’ New Diet Revolution (Atkins), Carbohydrate Addict’s diet, Sugar Busters, Weight Watchers, “Eat More Weigh Less” by Dean Ornish (Ornish), “Enter the Zone, A Dietary Roadmap” (Zone), “The LEARN Program for Weight Management 2000” (LEARN), Atkins for Life, South Beach, Dietary Approaches to Stop Hypertension (DASH), Best Life, New Glucose Revolution and the 2005 U.S. Department of Agriculture Food Guide Pyramid (2005 Food Guide Pyramid) [11,12,13,14]. These diets were reported to be insufficient in essential vitamins and minerals compared to recommended intakes [11,12,13,14].

Based on macronutrient composition, popular weight-loss diets that have been reported in the literature cover three types: High-Fat Low-Carbohydrate (HFLC), moderate-fat Balanced Nutrient Reduction (BNR) and low-fat/Very-Low-Fat (VLF) diets [12]. Nutritional analysis revealed nutrient deficits in these diets: HFLC diets such as *Atkins* fall short with respect to vitamin A, E, B_6_, thiamin, folate, calcium, magnesium, iron, potassium and dietary fiber; BNR diets (e.g., Weight Watchers) may not provide recommended intakes of vitamin B_12_, calcium, zinc, magnesium, iron and dietary fiber if dieters do not make proper food choices; VLF diets like Ornish are low in vitamin E, B_12_ and zinc [12]. The underlying philosophy of these weight-loss diets is to reduce total calories or caloric density [12]. There are diet plans that are designed with a different rationale for weight loss (e.g., via increased nutrient density or metabolic rate) or maintenance (e.g., via energy balance). Unfortunately, there are no published data about the nutritional adequacy of such diets. To fill the gap in this regard, in the present study we analyzed nutrient profiles of three popular plans that represent the following commercial diet types: hypocaloric vegan diets that promote nutrient density, high-animal-protein low-carbohydrate diets that aim to increase metabolic rate and weight maintenance diets that target energy balance. The objective of this study was to evaluate micronutrient sufficiency of three select diet plans representing aforementioned diet regimens. We hypothesized that micronutrient deficits were prevalent in these plans.

## 2. Methods

### 2.1. Selection of Weight-Loss Diet Plans

Selection of specific diet plans was established by a ranking based on number of reviews on Amazon.com. A search on Amazon.com was conducted on December 18, 2016. Of the top five diets (Table S1), numbers 1 and 2 were excluded because they were designed for rapid weight loss within a short period of time (10 days). Number 4 was excluded as a single set of seven-day meal menus was not representative of the entire diet plan. The two remaining plans, “Eat to Live-Vegan, Aggressive Weight Loss (ETL-VAWL)” and “Fast Metabolism Diet (FMD)”, were selected to represent two genres of commercial weight-loss diets, hypocaloric vegan and high-animal-protein low-carbohydrate diets [15,16]. In addition, a third diet plan, “Eat, Drink and Be Healthy (EDH)”, was included in the diet analysis to be representative of weight maintenance diets [17]. All three diet plans were summarized in Table 1.

Eat to Live-Vegan, Aggressive Weight Loss (ETL-VAWL): The Eat to Live diet is a rigorous six-week diet plan which can also be continued indefinitely and more flexibly as a “Life Plan”. It advises a plant-based diet with minimal consumption (but not necessarily absent) of animal products unless certain diet protocols are implemented. The Vegan Aggressive Weight Loss protocol (ETL-VAWL) was analyzed in this study and this diet protocol does not include any animal product. The six-week plan does not restrict raw or cooked non-starchy vegetables and calls for 1 cup of beans or legumes and 4 servings of fruit (but not fruit juice) daily. It limits starchy vegetables, grains, nuts, seeds and dried fruits and excludes refined grains, sugars, oils and dairy.

The ETL-VAWL diet attributes obesity and poor health to the overconsumption of nutrient-poor foods such as refined sugars, grains and oils [15]. In lieu of processed foods, the ETL-VAWL protocol advises dieters to increase nutrient density of the diet on a per-calorie basis and emphasize adequate intake of fruits and vegetables.

Fast Metabolism Diet (FMD): The goal of FMD is to maximize energy expenditure rather than restrict calories per se, though the diet is nevertheless hypocaloric [16]. The 28-day diet plan comprises four weekly cycles through three phases: (1) Phase 1 (“Unwind Stress”) emphasizes the consumption of high-glycemic index carbohydrates (rice, oatmeal), fruits (mangos, apples) and very low fat intake; (2) Phase 2 (“Unlock Fat Stores”) is a high-protein, low-carbohydrate, low-fat intervention based on consumption of lean protein (e.g., lean meats) and green, non-starchy vegetables; (3) Phase 3 (“Unleash the Burn”) encourages intake of plant-based fats (e.g., nuts, seeds, avocados, coconuts, olives) and marine polyunsaturated fats (e.g., long-chain omega-3 fatty acids from salmon), as well as low-glycemic index sources of carbohydrates such as berries and unrefined grains (excluding wheat and corn).

The FMD protocol encourages avoidance of wheat, corn, dairy, refined sugars, caffeine, alcohol, dried fruit, fruit juice, artificial sweeteners and “diet” products. Soy consumption is prohibited, except for vegans on Phase 2 of the diet.

Eat, Drink and Be Healthy (EDH): Unlike the other two weight-loss diet plans, the EDH plan is a lifestyle protocol based on the “Healthy Eating Pyramid” [17]. It recommends high intake of vegetables, fruits, nuts and legumes, frequent use of plant-derived oils (e.g., olive, canola, soybean) and moderate intake of fish, poultry and eggs. Calcium intake may be obtained from dairy or non-dairy sources and calcium supplementation is advised only if the diet is deficient. Moderate alcohol intake is allowed but intake of refined grains, red meat, potatoes, desserts and sugar-sweetened beverages is minimized on the EDH diet plan.

This diet plan embraces a bioenergetics paradigm (calories in = calories out for weight maintenance) but neither requires nor encourages strict measurement of portions or calorie intake. The weekly meal plans are based on a 2000-kilocalorie maintenance diet but are readily adjustable depending on weight goals.

### 2.2. Meal-Plan Analysis

Seven single-day menus of each diet were sampled to represent one week of menus on each diet regimen (*n* = 21 menus, 7 menus/diet). Sample menus were manually entered into the online nutrient tracker CRON-O-Meter (https://cronometer.com/). CRON-O-Meter is a web-based nutrition and biometric tracking application which features data on over 60 nutrients and contains more than 7500 foods in its databases [18]. CRON-O-Meter was chosen as a tool for nutrient analysis mainly because of its large food database including the U.S. Department of Agriculture (U.S. DA) National Nutrient Database for Standard Reference. Additional databases included in CRON-O-Meter are the Nutrition Coordinating Center Food and Nutrient Database (NCCDB), ESHA Database, Canadian Nutrient File (CNF) and the Irish Food Composition Database (IFCDB).

Food items in the CRON-O-Meter database that most closely matched menu descriptions (including preparation method and portion size) were entered for analysis. When pooled databases contained multiple listings for any food item, only one food item was selected following the priority order of U.S. DA > NCCDB > other databases (ESHA, CNF, IFCDB). Exact portions as listed in menus were entered. In cases where portions were not specified, the following criteria were applied to assign portions: when Nutrition Labeling and Education Act (NLEA) Servings were available in the CRON-O-Meter database, NLEA portions were used; otherwise standard unit values were selected on a discretionary basis (e.g., “orange juice” = 1 cup; “grapefruit” = 1 whole fruit). For mixed foods with accompanying recipes, 1 serving of each recipe was entered unless otherwise specified in menus.

Dietary Reference Intake (DRI) values for U.S. adult men aged 19–50 years were selected as reference values of nutrient adequacies in this analysis as they are less variable than those for females (e.g., variation by menopause, pregnancy and lactation) [19]. Amounts of macronutrients and 20 micronutrients in each diet plan were calculated. Given there were differences in calories between three diet plans, micronutrient content was further adjusted for a daily 2000-kcal intake. Both unadjusted and adjusted micronutrient levels of each diet plan were compared to DRIs for U.S. male adults.

This analysis assumed that each daily meal plan would be consumed with no deviation from food items and portions that were manually entered into the CRON-O-Meter software (100% compliance) and no other source of nutrients (e.g., dietary supplements) was consumed beyond food items listed in menus. The following nutrients were not included in the diet analysis: (1) vitamin A as the type of vitamin A (e.g., retinol versus carotenoids) translates into highly variable retinol activity equivalent values for different pro-vitamin A carotenoids, complicating assessment of micronutrient sufficiency by meal plan analysis; (2) choline, biotin, chromium, iodine and molybdenum as they are not consistently reported for foods in the CRON-O-Meter database; (3) sodium as meal plans do not always clearly define sodium content.

## 3. Results

The three diet plans varied in daily calories ranging from 1137 kcal to 2015 kcal and macronutrient content (Table 2). Specifically, the ETL-VAWL diet plan was lower in protein than the other two plans (51 g versus 85–90 g). As shown in Table 3, without adjustment for energy intake, the ETL-VAWL diet failed to provide 90% of recommended amounts (DRIs for U.S. male adults) for seven micronutrients including vitamin B_3_ (89% DRI), B_12_ (30% DRI), D (6% DRI), E (73% DRI), calcium (78% DRI), selenium (65% DRI) and zinc (75% DRI). The FMD diet was low (<90% of DRI) in B_1_ (83% DRI), D (5% DRI), E (83% DRI), calcium (46% DRI), magnesium (86% DRI) and potassium (71% DRI). The EDH diet met >90% DRIs for all analyzed micronutrients except vitamin D (28% DRI), calcium (67% DRI) and potassium (89% DRI).

After micronutrient content of each diet plan was adjusted for a 2000-kcal diet, all diet plans failed to provide adequate amounts of all 20 micronutrients (Table 4): the adjusted %DRI value for vitamin B_12_ on ETL-VAWL was 45%; FMD and EDH remained low (<90% DRI) in calcium (adjusted %DRI: 81% for FMD; 66% for EDH); Vitamin D was below the recommended daily amount (600 IU for U.S. adult men) across all three diets (9%, 9%, 28% DRI for ETL, FMD, EDH, respectively).

## 4. Discussion

Commercial weight-loss diet plans vary greatly, not only in energy content but also in macronutrient and micronutrient composition [11,13,14]. In the present analysis, protein content in the vegan diet plan (ETL-VAWL) was about 42% lower than in the other two plans (FMD and EDH), which is worth noting due to the role protein plays in preserving muscle mass. Although there are limited data on the risk of nutrient deficiencies among dieters, select weight-loss diets have been evaluated using self-reported dietary recalls or menu analysis. Dietary intakes collected by self-reported recalls are subject to underreporting especially among overweight and obese individuals. In addition, compliance issues often pose a challenge when using dietary recall data as a proxy of actual nutrient content in diet menus. Menu analysis is an objective method to investigate nutrient profiles of diet plans and is not subject to recall bias due to reliance on participants’ memory. In our menu analysis, out of twenty micronutrients that were assessed, 2–6 essential micronutrients were below 90% of the recommended intakes (DRIs for U.S. male adults) in the three diets. After micronutrient content was standardized to a 2000-calorie diet, vitamin D was substantially lower in all diets (9–28% male DRI of 600IU); in addition, B_12_ was low in ETL-VAWL (45% male DRI) and calcium in FMD (81% male DRI) and EDH (66% DRI).

For the twenty micronutrients that were analyzed in our study, recommended intakes for U.S adult women and men are the same for 11 vitamins and minerals (vitamin B_5_, B_6_, B_12_, folate, D, E, calcium, copper, selenium, potassium and phosphorus) [19]. The daily requirement of iron for pre-menopausal adult women is much higher than that for men (18 mg versus 8 mg) [19]. For the remaining micronutrients (vitamin B_1_, B_2_, B_3_, C, K, magnesium, manganese and zinc), female DRIs are lower than male values [19]. Given these differences between female and male DRIs, we performed additional analysis using DRIs for U.S. adult women. Without adjustment for energy intake, the following micronutrients did not meet 90% of the recommended intakes for women: (1) vitamin B_12_ (29% DRI), D (6% DRI), E (73%), calcium (78% DRI) and selenium (65% DRI) in ETL-VAWL; (2) vitamin D (5% DRI), E (83% DRI), calcium (46% DRI), iron (72% DRI) and potassium (71% DRI) in FMD; (3) vitamin D (28% DRI), calcium (67%) and potassium (89%) in EDH (data not presented). Compared to men, women, on average, have lower body weight and consume fewer calories regardless if on a weight loss or maintenance diet. On this basis women may take in proportionately less of these nutrients than men and therefore be at higher risk for micronutrient insufficiencies when following these diet plans. Future research is needed to assess differences in risk of nutrient insufficiencies by gender among dieters.

Our data are supportive of micronutrient deficits and questionable dietary quality reported in previous work [11,12,13,14]. Daily menus of four weight-loss diets (Atkins for Life, South Beach, DASH, Best Life) were previously analyzed for 27 micronutrients contents and reported to be consistently low in six micronutrients including vitamin B_7_ (biotin), D, E, chromium, iodine and molybdenum compared to the minimum Reference Daily Intake (RDI) levels determined by U.S. Food and Drug Administration [14]. Dietary quality of seven weight-loss plans (New Glucose Revolution, Weight Watchers, Atkins, South Beach, Zone, Ornish and 2005 Food Guide Pyramid) was assessed using the Alternate Healthy Eating Index (AHEI), a measure for cardiovascular risk reduction and all diets did not reach an optimal AHEI score [13]. In another trial, overweight or obese women were randomly assigned to one of four diet plans (Atkins, Zone, LEARN and Ornish) for 8 weeks and their dietary intakes were evaluated using 24-h recalls both at baseline and the end of intervention [11]. Significant differences in intake of macronutrients and micronutrients were reported between these diets. Specifically, all diet plans did not provide adequate amounts of essential vitamins and minerals, which translated to inadequate intakes (below the Estimated Average Requirement value; EAR) of 2–6 essential micronutrients among >25% of women at the end of the study [11]. Furthermore, compared to baseline, the risk of micronutrient inadequacy (below EAR) or lower intake (below the Adequate Intake; AI) after the 8-week intervention was significantly increased for several vitamins (vitamin C, E, B_12_, thiamin, folic acid) and minerals (calcium, iron, magnesium and zinc) among at least one diet group (*p* < 0.01) [11]. The 2015 Dietary Guidelines Advisory Committee (DGAC) report listed 10 “shortfall nutrients” including vitamins C, D, E, folate, calcium, magnesium and iron (for adolescents and premenopausal women) [20]. In a broader context, the United Nations declared 2016–2025 as a decade of action on nutrition and call for double duty actions to address both under- and over-nutrition including overweight, obesity and nutrition-related noncommunicable diseases (NCDs) [21]. In order to reduce the risk of micronutrient deficiencies while balancing macronutrient needs among dieters, micronutrient composition needs to be an indispensable component of overall nutrition quality of weight-loss diets [11,13,14].

A relevant question that needs to be asked is if people who are dieting can realistically achieve adequate nutrient intakes purely via food? Diet optimization research does not support the feasibility of achieving recommended nutrient levels via food only while following diet plans. Diet optimization was performed on four diet plans (Atkins for Life, South Beach, DASH, Best Life) to calculate the required calories in order to provide 100% RDIs for 21 and 27 essential micronutrients [14]. Original macronutrient composition (ratios) in each diet plan was kept unchanged during the analysis for 100% sufficiency [14]. In order to reach 100% RDI sufficiency for 21 essential micronutrients, the required daily calories were calculated to be 2425 kcal, 3175 kcal, 3300 kcal and 5000 kcal for the South Beach, Atkins, Best Life and DASH diets, respectively [14]. In order to meet 100% RDI for 27 micronutrients, these diets would need to provide unrealistically high calories ranging from 18,800 to 37,500 kcal daily [14]. In the present analysis, we calculated micronutrient content of each diet at a daily energy intake of 2000 kcal (nutrient density per 2000 calories). At a daily 2000-kcal intake, the three diets failed to provide sufficient amounts of at least two micronutrients (<90% DRI) and vitamin D was consistently below 90% DRI (600 IU) in all three plans (51–169 IU per 2000 calories). These data collectively suggest current diet plans have failed to provide sufficient amounts of essential micronutrients at realistic calorie intake, which can increase the risk of micronutrient deficiency in dieters.

Micronutrient deficits in weight-loss diets may create a vicious cycle among overweight and obese individuals: they are on diet in order to lose weight but cannot attain adequate amounts of essential nutrients by following diet plans; low intakes may translate into increased risk of being micronutrient deficient; these deficiencies are known as a causal factor to NCDs including obesity and related clinical conditions [10]. As an example, vitamin D and calcium are listed as two of “nutrients of public health concern” in the 2015 DGAC report due to consistent under-consumption across diverse demographic groups (ethnicity, age and gender) and the associated health impact [20]. The role of vitamin D and calcium in the prevention of low bone mass and associated complications (i.e. osteoporosis) has been well documented in the literature. Considering the number of Americans with osteoporosis (≥9.9 million) or low bone density (43.1 million) and the projected relevant health care cost (US$25.3 billion by 2025) [22], adequate intakes of vitamin D and calcium are necessary for the public including dieters to achieve overall wellness and manage associated disease burdens.

In order to help people achieve recommended intakes of nutrients, the American Medical Association announced its position of advising “all adults to take at least one multivitamin pill each day” [23] and the 2015 DGAC recommended dietary supplements as useful to fill nutrient gaps in the American diet [20]. The association between dietary supplement use and a lower prevalence of micronutrient insufficiency has been reported among children, adolescents, adults and the elderly [24,25,26,27]. Since there are many factors that influence nutritional status of an individual including, but not limited to, inherent biological factors, lifestyle (i.e., physical activity levels, dietary intakes), host conditions (i.e., morbidities and medications), socioeconomic status and geographical location [10,28], a holistic approach integrating dietary, lifestyle and behavioral interventions may help at-risk populations such as overweight and obese dieting individuals meet their nutritional needs.

There are several limitations in our diet analysis. First, assumptions were made for serving size when such information was missing or unspecified in the sample meal plans. In such cases, NLEA portions were used when available in the CRON-O-Meter database; otherwise standard unit values were selected on a discretionary basis. Second, our menu analysis assumed 100% compliance to diet menus and no other sources of nutrient intakes than menu items. Adherence to prescribed diet plans has been reported to be a major challenge for free-living individuals, especially long term [29]. When people follow these weight-loss diets for an extended period, sampled diet menus may not accurately represent actual nutrient intakes due to low compliance and/or intake of nutrients from other sources such as dietary supplements. Moreover, several nutrients including sodium, vitamin A, choline, biotin, chromium, iodine and molybdenum were not analyzed in the present study due to methodological challenges as described in the section of Methods. In addition, under-consumption of certain micronutrients over time may increase risk of deficiencies and influence health, which is not assessed in this study but is worth evaluating in future studies. Lastly, our analysis did not perform diet optimization to examine the calorie level needed to provide 100% DRI micronutrients, which is worth exploring in future analysis.

## 5. Conclusions

To our knowledge, this study is the first to analyze seven single-day menus and nutrient density to evaluate micronutrient profiles of commercial diet plans (ETL-VAWL, FMD and EDH) compared to DRIs for U.S. male adults. At a daily energy intake of 2000 kcal, the three diets did not provide recommended intakes (<90% DRI) of at least two micronutrients (vitamin D + calcium or B_12_) and one micronutrient (vitamin D) was below 600 IU (<30% DRI) across all diet plans. Our data complement and support previously published literature that micronutrient deficits are prevalent in weight-loss diet plans. More research is needed to find an optimal balance between macronutrient and micronutrient content in weight-loss diets in order to reduce risk of micronutrient deficiency among dieters. If not, adding an economically effective way of filling nutrient gaps, such as appropriate dietary supplements, should be considered.

## Figures and Tables

**Table 1:** Summary of three weight-loss diet plans for meal-plan analysis *.

	Eat to Live-Vegan, Aggressive Weight Loss (ETL-VAWL)	Fast Metabolism Diet (FMD)	Eat, Drink and Be Healthy (EDH)
**Philosophy**	Increase “nutrient density” (nutrients per calorie) of the diet to lose weight and improve health.	Increase “metabolic rate” to facilitate weight loss while improving body composition.	Achieve and maintain a healthy weight via consumption of a whole-food, plant-based diet (“Healthy Eating Pyramid”).
**Focal Foods**	Fruits, vegetables, legumes.	Phase 1: High-glycemic index carbohydrates (rice, oatmeal) and fruits (mangos, apples). Phase 2: high-protein, low-carbohydrate, low-fat foods (lean meats, green, leafy vegetables). Phase 3: plant-based fats (e.g., nuts, seeds, avocados, coconuts and olives), marine polyunsaturated fats (e.g., fatty fish), low-glycemic index fruits and vegetables.	Whole grains, fruits, vegetables, plant-derived oils, nuts, legumes.
**Limited Foods** **	Low-fiber, nutrient-poor, refined grains, added sugars, oils, animal foods, dairy products, fruit juices.	Wheat, corn, dairy, soy, refined sugar, caffeine, alcohol, dried fruit and fruit juices, artificial sweeteners and ‘diet’ foods.	Red meat, saturated fats, refined grains, potatoes, soft drinks, candies and desserts.
**Recommendations on Supplements** ***	Multi-vitamin, vitamin D, vitamin B_12_, zinc, iodine, plant-derived omega-3 fatty acids.	Multi-vitamin, essential fatty acids.	Multi-vitamin, fish oil (but whole fish is preferred), calcium (if diet is deficient), vitamin D and vitamin E.

* Adapted from [15,16,17]. ** ETL-VAWL: six-week plan eliminates dairy and animal foods, snacking, fruit juices and oils. The plan suggests a “90-percent rule” whereby the greater fraction of the diet comprises unprocessed plant foods with a 10% discretionary allowance. FMD: the plan encourages avoidance of ten items on the list. EDH: items listed are recommended to be “used sparingly”. *** ETL-VAWL strongly encourages fulfilling nutrient requirements via whole-food diet but recommends supplements to fill specific nutrient gaps if there is any. FMD recommends supplements based on “poor soil quality” and “occasional…poor food choices”. EDH recommends a multivitamin as it is generally safe and affordable.

**Table 2:** Macronutrient content of three weight-loss diet plans *.

Macronutrient	Eat to Live-Vegan, Aggressive Weight Loss (ETL-VAWL)	Fast Metabolism Diet (FMD)	Eat, Drink and be Healthy (EDH)
Energy (kcal)	1302 ± 298	1137 ± 228	2015 ± 134
CHO (g)	225 ± 45	116 ± 51	281 ± 16
Fiber (g)	49 ± 8	27 ± 10	40 ± 3
Sugars (g)	109 ± 27	41 ± 18	108 ± 28
Fat (g)	35 ± 16	43 ± 27	66 ± 13
Mono (g)	11 ± 7	19 ± 15	30 ± 8
Sat (g)	6 ± 3	10 ± 8	11 ± 2
Poly (g)	14 ± 7	10 ± 7	20 ± 7
*n*-3 (g)	2.6 ± 0.8	0.9 ± 0.4	2.8 ± 0.8
*n*-6 (g)	11.1 ± 5.8	8.4 ± 7.1	16.4 ± 7.6
Trans (g)	0 ± 0	0.1 ± 0.2	0.1 ± 0.1
Cholesterol (mg)	0 ± 0	225 ± 81	278 ± 157
Protein (g)	51 ± 15	85 ± 21	90 ± 18

* Macronutrient amounts were obtained based on seven single-day menus per diet plan. Values were expressed as mean ± Standard Deviation (SD). CHO: carbohydrate. Mono: mono-unsaturated fat. Sat: saturated fat. Poly: poly-unsaturated fat. *n*-3: omega-3 poly-unsaturated fat. *n*-6: omega-6 poly-unsaturated fat. *Trans*: *trans* fat.

**Table 3:** Micronutrient content of three weight-loss diet plans compared to Dietary Reference Intake (DRI) values for U.S male adults *.

Micronutrient	Eat to Live-Vegan, Aggressive Weight Loss (ETL-VAWL)		Fast Metabolism Diet (FMD)		Eat, Drink and Be Healthy (EDH)	
	Amount	%DRI (%)	Amount	%DRI (%)	Amount	%DRI (%)
B1 (mg)	1.7 ± 0.5	142 ± 42	1.0 ± 0.3	83 ± 25	2.0 ± 0.4	170 ± 34
B12 (mcg)	0.7 ± 1.0	30 ± 42	3.5 ± 2.9	146 ± 121	2.9 ± 3.2	122 ± 132
B2 (mg)	1.5 ± 0.4	115 ± 31	1.4 ± 0.5	108 ± 38	2.0 ± 0.6	154 ± 44
B3 (mg)	14.3 ± 3.2	89 ± 20	24.1 ± 8.1	151 ± 51	28.1 ± 10.5	176 ± 65
B5 (mg)	5.4 ± 1.4	108 ± 28	4.7 ± 1.0	94 ± 20	6.2 ± 1.8	124 ± 35
B6 (mg)	2.7 ± 0.6	208 ± 46	2.5 ± 0.5	192 ± 38	3.0 ± 0.8	231 ± 65
Folate (mcg)	832 ± 194	208 ± 49	444 ± 130	111 ± 33	644 ± 97	161 ± 24
Vitamin C (mg)	447 ± 180	497 ± 200	209 ± 109	232 ± 121	267 ± 123	297 ± 136
Vitamin D (IU)	33.3 ± 39.7	6 ± 7	32.1 ± 42.0	5 ± 7	169.8 ± 300.2	28 ± 50
Vitamin E (mg)	11.0 ± 3.6	73 ± 24	12.4 ± 10.5	83 ± 70	14.7 ± 3.7	98 ± 25
Vitamin K (mcg)	603 ± 269	503 ± 224	440 ± 330	367 ± 275	264 ± 80	220 ± 67
Calcium (mg)	782 ± 305	78 ± 31	461 ± 176	46 ± 18	668 ± 210	67 ± 21
Copper (mg)	2.6 ± 0.7	289 ± 78	1.5 ± 0.5	167 ± 56	2.1 ± 0.3	229 ± 33
Iron (mg)	17.2 ± 3.9	215 ± 49	12.9 ± 2.4	161 ± 30	19.3 ± 5.4	242 ± 68
Magnesium (mg)	484 ± 79	115 ± 19	361 ± 86	86 ± 20	492 ± 47	117 ± 11
Manganese (mg)	5.5 ± 1.1	239 ± 48	3.3 ± 1.2	143 ± 52	7.0 ± 2.2	303 ± 95
Phosphorus (mg)	1099 ± 265	157 ± 38	1175 ± 175	168 ± 25	1548 ± 227	221 ± 32
Potassium (mg)	4861 ± 526	103 ± 11	3330 ± 457	71 ± 10	4188 ± 804	89 ± 17
Selenium (mcg)	35.7 ± 20.2	65 ± 37	125.5 ± 41.3	228 ± 75	127.1 ± 23.7	231 ± 43
Zinc (mg)	8.3 ± 2.0	75 ± 18	10.0 ± 3.5	91 ± 32	10.6 ± 1.9	96 ± 17

* Micronutrient amounts were obtained based on seven single-day menus per diet plan. Values were expressed as mean ± Standard Deviation (SD). DRI values for U.S. adult men (age 19–50 years) were used to calculate %DRIs for micronutrients except for magnesium. The DRI value for U.S. adult men (age 31–50 years) was used to calculate %DRI for magnesium. Values of %DRIs were expressed as mean ± Standard Deviation (SD).

**Table 4:** Nutrient density * (per 2000 calories) in three weight-loss diet plans compared to Dietary Reference Intake (DRI) values for U.S male adults.

Micronutrient	Eat to Live-Vegan, Aggressive Weight Loss (ETL-VAWL)		Fast Metabolism Diet (FMD)		Eat, Drink and be Healthy (EDH)	
	Nutrient Density *	Adjusted % DRI (%) **	Nutrient Density *	Adjusted %DRI (%) **	Nutrient Density *	Adjusted %DRI (%) **
B1 (mg)	2.6	218	1.8	147	2.0	169
B12 (mcg)	1.1	45	6.2	257	2.9	121
B2 (mg)	2.3	177	2.5	189	2.0	153
B3 (mg)	22.0	137	42.4	265	27.9	174
B5 (mg)	8.3	166	8.3	165	6.2	123
B6 (mg)	4.1	319	4.4	338	3.0	229
Folate (mcg)	1278	320	781	195	639	160
Vitamin C (mg)	687	763	368	408	265	295
Vitamin D (IU)	51.1	9	56.5	9	169	28
Vitamin E (mg)	16.9	113	21.8	145	14.6	97
Vitamin K (mcg)	926	772	774	645	262	218
Calcium (mg)	1201	120	811	81	663	66
Copper (mg)	4.0	444	2.6	293	2.0	227
Iron (mg)	26.4	330	22.7	284	19.2	240
Magnesium (mg)	743	177	635	151	488	116
Manganese (mg)	8.4	367	5.8	252	6.9	301
Phosphorus (mg)	1688	241	2067	295	1537	220
Potassium (mg)	7467	159	5858	125	4158	88
Selenium (mcg)	54.8	100	220.8	401	126.2	229
Zinc (mg)	12.7	116	17.6	160	10.5	96

* Micronutrient amounts were obtained based on seven single-day menus per diet plan and mean values were adjusted for a daily energy intake of 2000 kcal. Nutrient density (unit of nutrient per 2000 kcal) = (mean nutrient content/energy content of diet plan) × 2000. ** DRIs for U.S. adult men (age 19–50 years) were used to calculate adjusted %DRIs. Adjusted %DRI = (nutrient density/DRI) × 100.

## Supplementary Materials

The following are available online at http://www.mdpi.com/2072-6643/10/1/108/s1, Table S1: Top five most reviewed weight-loss diet books on Amazon.com.
