# Supplementary material for: Micronutrient Gaps in Three Commercial Weight-Loss Diet Plans
Source: Nutrients. 2018 Jan 20;10(1):108. doi: 10.3390/nu10010108 (PMC5793336; doi:10.3390/nu10010108)
Supplement: Supplementary file 1 [file nutrients-10-00108-s001.pdf]

Supplementary Table 1: Top five most reviewed weight-loss diet books on Amazon.com.

Ranking *	Weight-loss Diet Books	No. of Reviews on Amazon.com
1	Smith JJ. 10-Day Green Smoothie Cleanse. 2014.	7613
2	Davis W, Tom Weiner. Wheat Belly: 10-Day Grain Detox: Reprogram Your Body for Rapid Weight Loss and Amazing Health. 2011.	5669
3	Fuhrman J, Oz M. **Eat to Live: The Amazing Nutrient-Rich Program for Fast and Sustained Weight Loss**. 2005.	4510
4	Hartwig D, Hartwig M. It Starts With Food: Discover the Whole30 and Change your Life in Unexpected Ways. 2014.	3306
5	Pomroy H, Eve Adamson E. **The Fast Metabolism Diet**: Eat More Food and Lose More Weight. 2013.	3228

* A search on Amazon.com was conducted on December 18, 2016 and rankings were based on number of reviews on Amazon.com.
